# The prevalence of hearing impairment in infants and children with down syndrome a cross sectional study in a Tertiary Care Center

**DOI:** 10.1038/s41598-025-90500-7

**Published:** 2025-03-04

**Authors:** Sohier Yahia, Marwa Metawea, Ahmed Megahed, Wessam ELshawaf, Yahya Wahba, Ranim Mahmoud

**Affiliations:** 1 Department of Pediatrics, Faculty of Medicine, Mansoura University, Mansoura, Egypt; 2 Faculty of Medicine, Mansoura University, Mansoura, Egypt; 3 Department of Audiology, Faculty of Medicine, Mansoura University, Mansoura, Egypt

**Keywords:** Prevalence, Down syndrome, Hearing, Impairment, Otitis media, Genetics, Clinical genetics

## Abstract

Down syndrome is the most common chromosomal disorder in humans. Hearing impairment is a common feature of Down syndrome. To assess the prevalence of hearing impairment in children with DS attending the Mansoura University Children’s Hospital. The study is a descriptive cross-sectional study on 170 pediatric patients with genetically confirmed DS. Patients were recruited from the genetic outpatient clinic of the Mansoura University Children’s Hospital from October 2021 to October 2022. All infants and children were subjected to full history taking, and a lateral X-ray on the nasopharynx with open mouth and extended neck. The ears were examined and cleared from accumulated cerumen if present. The presence of middle ear pathology was assessed through an otoscopic examination of the tympanic membrane. Eustachian tube dysfunction and otitis media with effusion (OME) were assessed using tympanometry. Appropriate hearing tests including pure tone audiometry (PTA) and auditory brain stem response (ABR) were used. Conductive hearing loss (CHL) was observed in 48.8% of studied children with DS and 4.1% had sensorineural hearing loss (SNHL). Among patients with hearing impairment, 86.5% had bilateral affection. The severity of hearing loss was mild in 59.1% of patients with CHL and 71.4% with SNHL. Of the patients with CHL, HL remained stationary in 45.8%, regressed from moderate to mild HL in 15.7%, and normalized in 38.6%. SNHL was permanent in all 7 affected patients with a stationary course in 4 and a progressive nature in 3. OME, upper respiratory tract infections (URTI), and adenoid enlargement were commonly associated with CHL in infants and children with DS. Most children with DS have bilateral and mild hearing loss. HL is mostly conductive. Otitis media with effusion, adenoid enlargement, and recurrent upper RTI are common in patients with conductive hearing loss. Hearing assessment should be considered in all infants and children with Down syndrome.

## Introduction

Down syndrome (DS) is the most common chromosomal trisomy in newborns and it is the most common genetic cause of intellectual disability^1^. The reported incidence of DS is about 1/700 births worldwide^2–4^ and 1/600 births in Egypt^5,6^. Down syndrome is a chromosomal disorder due to the presence of an extra copy of chromosome 21 and results from non-disjunction of chromosome 21 in 95% of individuals during maternal meiosis I (66%) or meiosis II (21%); paternal meiosis I (3%) or meiosis II (5%). Translocation, usually t (14;21) or t (21;21), accounts for 3% of affected individuals, and mosaicism accounts for 2%^4^.

Patients with DS have a variety of physical and developmental characteristics including learning disabilities, craniofacial abnormality, and hypotonia in early infancy. DS patients have distinct physical features like a small chin, upward slanted eye, poor muscle tone, a flat nasal bridge, a single palmer crease, and a protruding tongue due to a small mouth. Other features include abnormal fingerprint patterns and short fingers. Some patients with DS are affected by variant phenotypes including atrioventricular septal defects (AVSD), leukemia (both acute megakaryoblastic leukemia (AMKL) and acute lymphoblastic leukemia (ALL)), Alzheimer’s disease and hyperactivity disorder^7^.

Hearing impairment is a common problem among children with DS. Studies reported that about 60–80% of children with Down syndrome experience some degree of hearing loss^8,9^. Conductive hearing loss (CHL) is the most common type among children with DS and is often caused by chronic otitis media. CHL may also occur as a result of cerumen impaction in stenotic ear canals, eardrum perforation, retraction pockets, cholesteatoma, mastoid abnormalities, and abnormalities of the ossicular chain such as the structure of the incudomalleolar joint and /or incudostapedial joint^10^. Sensorineural hearing loss can be also seen in 10–20% of children with DS with current research showing significant inner ear abnormalities on computed tomography scans^11^.

Several factors can contribute to hearing loss in patients with DS. Otitis media with effusion (OME) is a highly prevalent condition in children with DS and the most common cause of CHL^12^. Several risk factors may contribute to OME in these children, such as immune deficiency with disturbances in T and B lymphocyte function, mid-facial hypoplasia, a crowded nasopharynx, hypotonia of the tensor veli palatine muscle, and a small easily collapsible Eustachian tube^13^. A narrow internal auditory canal has been identified in nearly 25% of individuals with DS^14^. Inner ear malformations, primarily in the vestibular organ, were documented in approximately 75% of patients with DS^14^. Even mild hearing loss can lead to delay in acquiring speech, especially in children with developmental disorders such as DS^11^.

Hearing impairment creates challenges for parents and clinicians because it can be difficult to diagnose. Critical management decisions are required to optimize speech development and social interaction^15^. Down Syndrome Medical Interest Group recommended annual hearing screening in preschool children with DS because of the difficulty in establishing the diagnosis and the potential long-term consequences of hearing impairment^16^. Treatment of OME aims to improve hearing and prevent long-term complications such as hearing loss and speech delay. Oral antibiotic treatment, antihistamines, decongestants, oral, and nasal corticosteroid therapy have little impact on retro tympanic effusion and do not improve hearing^17^.

Pressure equalizing tubes (PETs) are recommended as the first therapeutic approach in patients with DS. A systematic review including 20 studies reported that PET is the best treatment for OME in patients with DS with the least morbidity^18^. However, Mesolella et al., reported that ventilation tubes are less effective in treating OME in patients with DS due to difficult placement and the high complication rate as persistent otorrhea^19^. Fermo et al. recommended Eustachian tube insufflation with thermal water as an effective management of OME in patients with DS^20^.

The study aims to assess the prevalence of OME, permanent and transient hearing loss in children with DS attending Mansoura University Children’s Hospital.

## Patients and methods

A single-center descriptive cross-sectional study was conducted on 170 pediatric patients with genetically confirmed DS younger than seven years. This age cut-off was selected due to the high prevalence of hearing disorders in this age group. Patients were recruited from the genetic outpatient clinic at Mansoura University Children’s Hospital from October 2021 to October 2022. The audiological assessment was done at the Audiology Department of Mansoura University Hospital. Written informed consents were obtained from the parents of enrolled patients. The study protocol was approved by the Institutional Research Board (IRB: MS.20.03.1069) of Mansoura University Faculty of Medicine.

The diagnosis of DS was confirmed with karyotyping. The patients included in the study aged from birth to seven years as this age showed a high prevalence of hearing disorders. The exclusion criteria were respiratory tract infection at the time of audiological assessment; and other risk factors for hearing impairment such as congenital infections, prematurity, low birth weight, ear injuries, and ototoxic drug intake.

Infants and children included in the study were subjected to full history taking including maternal history, history of recurrent ear infection, and recurrent upper respiratory tract infections (URTI) such as sinusitis, pharyngitis, and otitis media. The incidence of URTI per year was questioned. Acute otitis media is considered recurrent if it occurs more than three times in six months (or four times in one year). Respiratory tract infections are considered recurrent when they occur more than 5times /year^21,22^. The ears were examined and cleared from accumulated cerumen if present. The presence of middle ear pathology was assessed through an otoscopic examination of the tympanic membrane. Eustachian tube dysfunction and OME were assessed using tympanometry. Tympanometry measures the mobility of the tympanic membrane and ossicular chain on changing the air pressure in a sealed external auditory canal using low-frequency probe tone (226 Hz) from − 400 to + 200 mmH2O. The results were recorded in a graph called a tympanogram. Type A: normal values when the peak pressure is between + 50 to – 100 daPa. Type B: flat tympanogram denotes otitis media with effusion. Type C (< -100): The tympanogram has a peak displaced towards negative pressure that occurs with ET dysfunction^23^. Lateral X-ray on the nasopharynx with open mouth and extended neck was obtained in patients with symptoms suggestive of adenoid enlargement. We used the adenoid-to-nasopharyngeal (A/N) ratio described by Fujioka et al. which is a ratio of the measurement of the adenoid tissue and the nasopharyngeal aperture. A/N ratio > 0.8 is an indicator of enlarged adenoids^24,25^.

Appropriate hearing tests were performed including Auditory Brain Stem Response (ABR) for infants and children younger than 5 years and pure tone audiometry (PTA) for those older than 5 years^26^. Conductive hearing loss was diagnosed when the pure tone air conduction average threshold was elevated while the bone conduction average threshold was normal. The difference between them is called the air-bone gap. Sensorineural hearing loss was diagnosed when air conduction and bone conduction thresholds were elevated with no air-bone gap. The tested frequencies were from 250 to 8000 Hz for air conduction using headphones and from 500 to 4000 Hz for bone conduction using a bone vibrator. Monaural pure tone average in the speech frequencies (500, 1000 and 2000 Hz) and according to the resultant levels: {normal hearing (-10 to 25 dBHL), mild hearing loss (26 to 40 dBHL), moderate hearing loss (41 to 55 dBHL), moderately severe hearing loss (56 to 70 dBHL), severe hearing loss (71 to 90 dBHL) and profound hearing loss (> 90 dBHL)^27^. Click ABR threshold was established as the minimum intensity at which wave V peak could be identified; normal hearing was taken as ≤ 40dBnHL^28^.

If patients had symptoms or signs suggestive of URTI, the audiological assessment was postponed for at least 2 weeks after treatment. Furthermore, newborn hearing screening and ABR test results at 3 months of age were available for 34 included patients. These test results were collected and analyzed. All tests were performed according to the relevant guidelines and regulations.

### Statistical analysis

The collected data were coded, processed, and analyzed using the SPSS (Statistical Package for Social Sciences) version 27 for Windows^®^ (IBM SPSS Inc, Chicago, IL, USA). Data were tested for normal distribution using the Shapiro Walk test. Qualitative data were represented as frequencies and relative percentages. Quantitative data were expressed as mean ± SD (Standard deviation) as well as median. Chi-Square test for comparison of categorical variables of 2 or more groups, Fischer Exact test was used as a correction for the Chi-Square test when more than 25% of cells count less than 5 in 2*2 tables. For all tests, the level of significance was tested, expressed as the probability of (p-value), and the results were explained as significant if p value was ≤ 0.05.

## Results

**Fig. 1:**
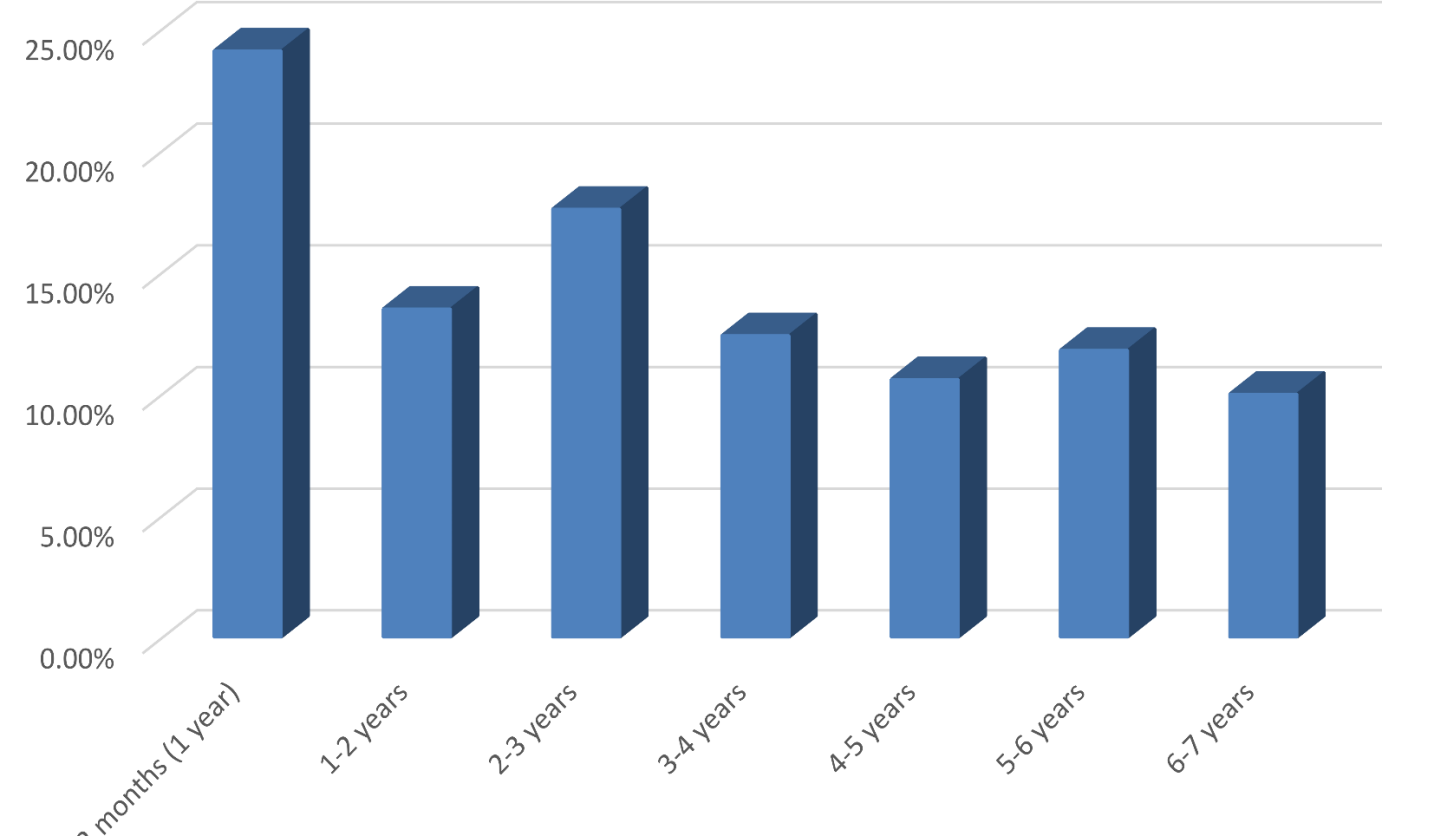
Age group distribution of the Down syndrome children.

The study included 170 patients with a mean age of 39.58 ± 26.43 months. Of the studied patients, 63.5% were males. Infants < 12 months represented 24.1% of the study population. Age distribution of the study cohort is shown in (Fig. 1). History of repeated URTI was reported in 62.9%. Lateral x-ray of the nasopharynx depicted adenoid hypertrophy in 53.5% of cases. Tympanometry was normal in 47.1% (80/170) of patients, whereas 35.8% (61/170) had OME and 17.1% (29/170) had Eustachian tube dysfunction.

An audiological assessment was done for all cases. Auditory brain stem response test was performed in 136 (80%) of cases and pure tone audiometry was performed in 34 (20%) of cases.

**Fig. 2:**
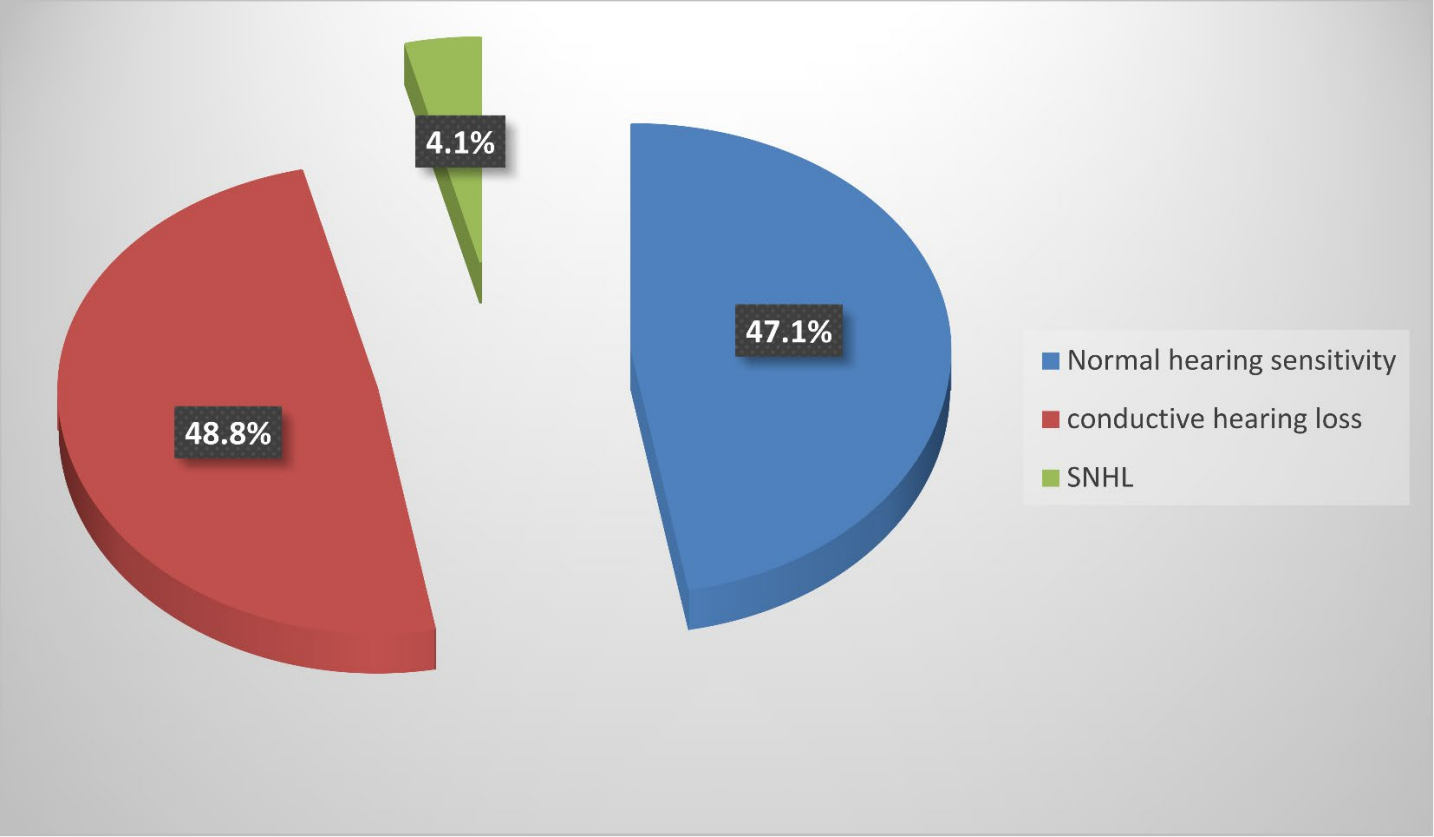
Overall hearing assessment in DS children.

**Fig. 3:**
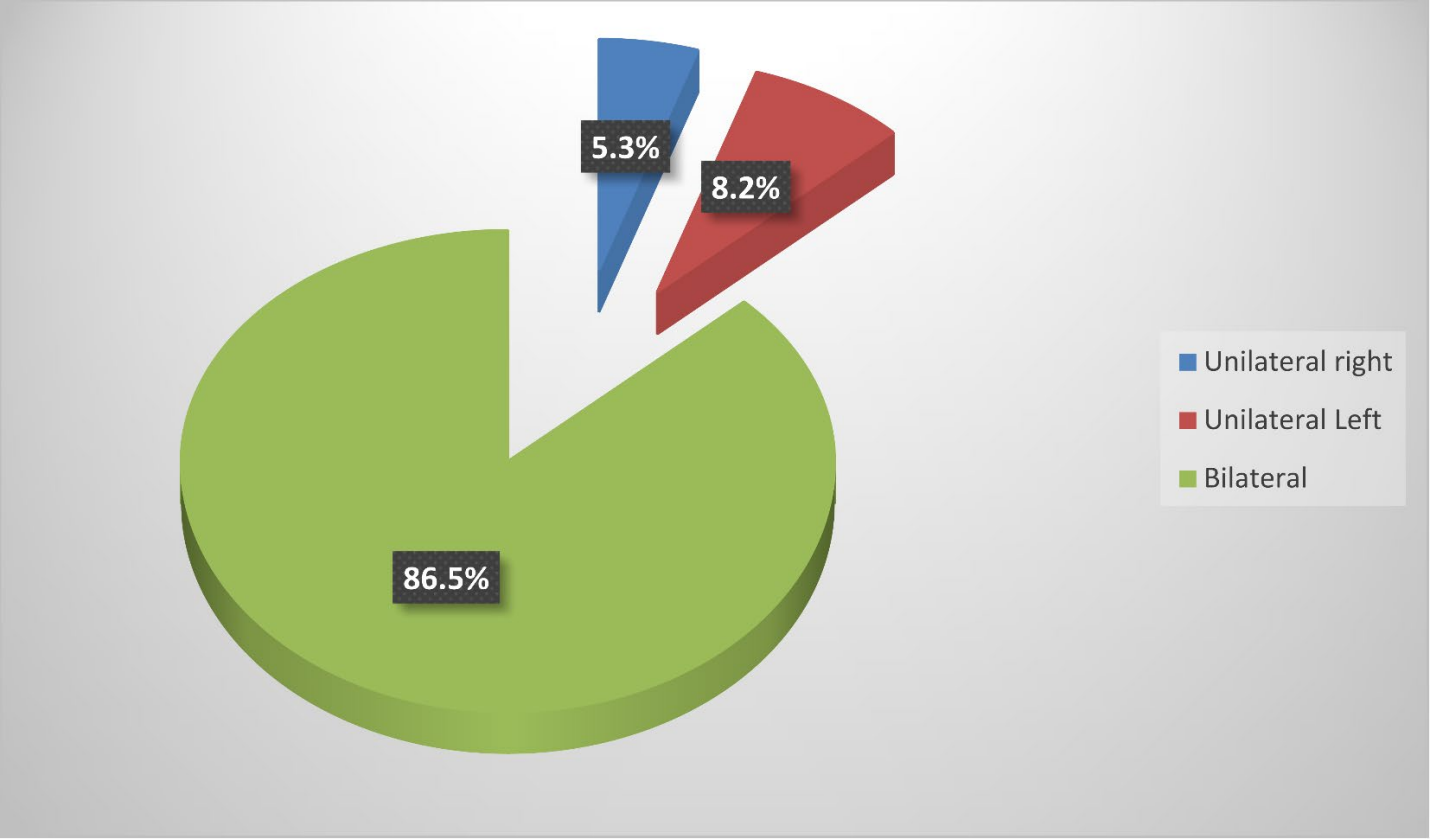
Side affection of hearing loss in DS children.

Among the study group, 48.8% (83/170) had CHL, and 4.1% (7/170) had sensorineural hearing loss (SNHL). We did not find cases of mixed hearing loss. Most patients (86.5%, 67/90) with hearing impairment, had bilateral affection. Hearing loss was mild in most affected patients, 59.1% (49/83) of patients with CHL and 5 of 7 patients with SNHL (Figs. 2 and 3). Patients with CHL were more likely to have a history of URTI and abnormal tympanometry (*P* < 0.001) (Table 1).

**Table 1:** Follow up of Down syndrome children with conductive hearing loss after completion of treatment and sensorineural hearing loss six months after the first assessment.

	Results of test			Test of significance
	Normal hearing sensitivity (*n* = 80)	Conductive hearing loss (*n* = 83)	SNHL (*n* = 7)	
Otitis media				
No	76 (95%)	26 (31.3%)	7 (100%)	MC = 18.689 *P* < 0.001*
Yes	4 (5%)	57 (68.7%)	0 (0%)	
Upper respiratory tract infection (URTI)				
No	49 (61.3%)	7 (8.4%)	7 (100%)	χ2 = 26.840 *P* < 0.001*
Yes	31(38.7%)	76 (61.9%)	0 (0%)	
Adenoid enlargement				
No	48 (60%)	25 (30.1%)	6 (85.7%)	MC = 19.140 *P* < 0.001*

Ninety patients were involved in a follow-up of 6 months after the initial assessment, CHL improved in 54.2% (45/83) of affected patients including hearing normalization in 32/45 (71.1%) and regression from moderate to mild hearing impairment in 13/45 (28.9%). In contrast, patients with SNHL had a stationary or progressive course in (57.1%) and (42.9%) of patients, respectively (Table 2).

**Table 2:** Follow up of Down syndrome children with conductive hearing loss after completion of treatment and sensorineural hearing loss six months after the first assessment.

Items	DS children with hearing loss *n* = 90	
	Number	Percent (%)
Conductive hearing loss	83	
Stationary	38	45.8
Improved (Transient hearing loss)	45	54.2
Normalization of hearing	32	71.1
Regressed from moderate to mild	13	28.9
Sensorineural hearing loss (permanent hearing loss)	7	7.8%
Stationary	4	57.1
Progressive	3	42.9

Categorical data expressed as Number (%).

The newborn hearing screening was available for 34 included infants. Of those, 22 (64.7%) passed the screening test bilaterally, 8 (23.5%) were unilaterally referred and 4 (11.8%) were bilaterally referred. The Auditory Brain stem response test was done in 34 infants 3 months after the newborn hearing screening. Test results were normal in 76.5% of cases, while (23.5%) had CHL. Hearing loss was mostly mild (75%) and bilateral (62.5%) (Table 3).

**Table 3:** Results of the auditory brain stem response test which was done 3 months after the newborn hearing screening in Down syndrome children.

Items	DS children	
	Number	Percent (%)
Normal	26	76.5
CHL	8	23.5
Degree of CHL		
Mild	6	75
Moderate	2	25
Side of affection		
Unilateral	3	37.5
Bilateral	5	62.5

Categorical data expressed as Number (%).

## Discussion

Down syndrome is among the most prevalent genetic disorders worldwide. Facial dysmorphic features, congenital heart defects, and hypotonia characterize it. In addition, it is associated with an increased frequency of hematological malignancies, autoimmune disorders, and recurrent infections mostly respiratory tract infection (RTI) and OME, suggesting dysfunction of the humoral immune response^29^.

Hearing loss is common in children with DS with a reported incidence as high as 78%, with a majority exhibiting CHL. Conservative estimates of congenital hearing loss in children with DS range from 15 to 20%^30^.

This study evaluated the prevalence of hearing impairment in infants and children with DS attending the Mansoura University Children’s Hospital. Notably, it is the first study on the hearing status of Egyptian children with DS across the pediatric age range from birth to seven years.

An auditory brain stem response test was performed in 80% of cases as it can estimate the hearing sensitivity thresholds in individuals unable to perform traditional behavioral audiometry^31^. Our study reported that 52.9% of infants and children with DS had hearing loss, with most (48.8%) of them having CHL and 4.1%had SNHL. Our findings align with the exciting literature. Pradilla et al.^32^ reported that 42.5% of Colombian children aged 6–18 years with DS had hearing loss, with 75% having CHL. Only one patient in that study had audiometry results consistent with SNHL. Our study found a higher rate of hearing loss in patients with DS compared to previous studies from China and Norway, which reported rates of 36% and 35%, respectively^33–35^. Park et al.,^36^ reported that the most frequent type of hearing loss in 344 newborns with DS was mild bilateral conductive loss, secondary to OME in 90%. Heß et al.^37^ found that 8.6% of DS patients had isolated SNHL.

Our study revealed that most infants and children with hearing impairment had mild and bilateral affection in CHL and SNHL. Basonbul et al.^38^ showed that out of 60 evaluated patients with DS aged 8 years or younger, 39/60 (65%) had hearing loss with variable degrees of severity. Nightengale et al.^39^ found that nearly 25% of patients with DS with a mean age of 5.99 ± 4.88 years had permanent hearing loss, mostly CHL 38%, with bilateral affection in 75%. Abnormal tympanograms were also identified in nearly 40% of patients; another 18% had results suggesting patent pressure equalization tubes.

The present study found that 61.9% of DS infants and children with CHL had a history of recurrent URTI. We found that 53.5% of DS patients had adenoid enlargement. Patients with CHL were more likely to have adenoid enlargement than SNHL (69.9% vs. 14.3% *p* < 0.001). Tympanometry was normal in 47.1% of patients, whereas 35.9% had OME and 17.1% had ETD. Patients with OME were treated with oral antibiotics, antihistamines, and decongestants. Patients were referred for PETs in case of failure of medical treatment. This is comparable to Austeng et al.‘s study^33^, which reported OME among 38% of DS children aged 8–9 years. Also, Martin et al.^40^ reported that 52.3% of DS infants had either OME or ventilation tubes at the time of their evaluation.

During the follow-up of cases with CHL, most infants and children improved (54.2%), while cases with SNHL remained stationary (57.1%) or progressed (42.9%). Similarly, Kreicher et al., 2018 found that hearing outcomes (hearing improved, unchanged, or worse) were influenced by the hearing loss type (*P* < 0.001) and type of tympanogram (*P* < 0.001). CHL, absence of cholesteatoma, and early placement of ear tubes before 2 years correlated with hearing improvement, Conversely, SNHL and mixed hearing loss were associated with progressive decline. More than one-half (51.8%; *n* = 912) of the ears of DS patients with hearing loss had a diagnosis of eustachian tube dysfunction. Patients with type B tympanogram showed significant improvement in the mean of PTA over time compared to those with type A tympanogram (*p* < 0.001)^8^.

The present study demonstrated that 64.4% of patients passed neonatal hearing screening bilaterally, 23.5% had unilaterally referred and 11.8% had bilaterally referred. This was comparable to a study including 232 newborns with DS of whom 69.9% passed newborn hearing screening^38^. In a study by Paulson et al.^15^, newborn hearing screening (NBHS) data of patients with DS who underwent PET placement was available in 89/102 patients. Of those, 56% (50/89) had normal hearing screens at birth and 44% initially failed their NBHS. The high initial pass rate suggests that the NBHS alone is not predictive of patients who may develop OME. Furthermore, it highlights the need for continued hearing screening of children with DS throughout development.

Yüksel et al.^41^ reported that newborn babies with DS had statistically higher rates of failing the initial and repeated hearing screening test by ABR than the control group (*p* < 0.001). Basonbul et al.,^38^ also reported a high failure rate of the newborn hearing screening test in patients with DS. Park et al., reported that 26% of patients with DS could not pass the initial hearing screening test and 43% of patients who passed the initial screening test developed CHL. These findings suggest that Patients with DS should be followed up even with a normal newborn hearing screening test^36^.

Our study included newborn screening (NBS) hearing results for patients with DS and auditory brainstem response (ABR) testing follow-up. Also, it is the first study to evaluate the correlation between type of hearing loss and OME, respiratory tract infection, and adenoid enlargement in infants and children with DS. However, our study has some limitations. The study population does not represent a community sample of children with DS, but rather a selected group of patients enrolled during the multidisciplinary care of patients with DS. Therefore, selection bias can affect the study results. Second, there is limited information regarding the audiologic interventions and treatment that could have affected the study outcomes. Our study findings highlight the importance of increasing social awareness about early diagnosis and referral for children with DS who have associated hearing problems. Early audiological interventions can help improve their speech-language development.

## Conclusion

Most infants and children with Down syndrome have bilateral and mild degree hearing loss, mostly conductive. OME, adenoid enlargement, and recurrent URTI are commonly associated with CHL. So, hearing assessment should be done in all infants and children with Down syndrome. Patients with DS should be followed up even with a normal newborn hearing screening test.

## Data Availability

The datasets used and/or analysed during the current study available from the corresponding author on reasonable request.
